# Real-world outcomes following adjuvant chemotherapy for resected pancreatic cancer in a centralised oncology service

**DOI:** 10.1038/s41416-026-03341-0

**Published:** 2026-02-25

**Authors:** Jessica Hale, Timothy Gilbert, Martyn Stott, Philip Whelan, Richard Jackson, Helen Wong, Marie McKay, Paula Ghaneh, Christopher Halloran, Olusola Faluyi, Declan Dunne, John P. Neoptolemos, Daniel Palmer

**Affiliations:** 1 The Clatterbridge Cancer Centre, Liverpool, UK; 2 Department of Molecular and Clinical Cancer Medicine, University of Liverpool, Liverpool, UK; 3 Liverpool University Hospitals NHS Foundation Trust, Liverpool, UK; 4 Liverpool Clinical Trials Centre, University of Liverpool, Liverpool, UK; 5 Liverpool Experimental Cancer Medicine Centre, University of Liverpool, Liverpool, UK; 6 Department of General, Visceral and Transplantation Surgery, Heidelberg University Hospital, Heidelberg, Germany

**Keywords:** Surgical oncology, Chemotherapy

## Abstract

**Background:**

Pancreatic cancer remains a significant challenge to diagnose and treat, with considerable regional variation in management and outcomes. This study aimed to evaluate real-world outcomes of patients receiving adjuvant treatment for pancreatic cancer at a single centre in Northwest England over an 11-year period.

**Methods:**

Data were collected retrospectively on all patients who underwent surgery for pancreatic ductal adenocarcinoma between 2009 and 2020. Collected data included patient demographics, surgical details and adjuvant treatment received, including number of chemotherapy cycles and dose reductions.

**Results:**

30-day/inpatient mortality was low (2.4%). Adjuvant chemotherapy delivery rates were high (82%) with 67.4% of patients completing the intended number of cycles. There was no additional survival benefit for patients who started chemotherapy within 8 weeks post-surgery compared to those who began later. Dose reductions did not impact survival, provided patients completed the full course of treatment (mOS 27.5 months vs. 28.5 months; HR 1.14, 95% CI 0.76–1.70 *p* = 0.513). Following centralisation of care, a greater proportion of patients commenced adjuvant treatment (86% vs 69% *p* < 0.05).

**Conclusion:**

A high proportion of patients received adjuvant treatment, with a centralised clinic model leading to increased rates of adjuvant chemotherapy delivery. Completion of the full chemotherapy course was more critical than dose intensity. Larger prospective studies are needed to investigate the factors contributing to regional variations.

## Introduction

Pancreatic ductal adenocarcinoma (PDAC) remains a devastating malignancy and a leading cause of cancer death worldwide [1, 2]. Overall, 5-year survival for all patients has improved from less than 5% to 13%, although this progress is largely due to developments in technical surgery and the use of adjuvant systemic chemotherapy [3, 4]. Unfortunately, only a minority of patients will present with operable disease. Randomised controlled trials show systemic treatment with adjuvant chemotherapy significantly prolongs overall and disease-free survival, compared to surgery alone in resectable pancreatic cancer [4]. This, along with the high post-operative recurrence rates, highlights the systemic nature of pancreatic cancer, even when the disease initially presents as resectable and localized. Such is the importance of systemic therapy that operating without it could be considered futile in achieving a long-term survival outcome [2, 4]. As a result, every effort should be made to maximise the number of patients receiving chemotherapy as part of their management plan.

Common adjuvant chemotherapy regimens for PDAC include gemcitabine [5, 6], 5-FU [7], gemcitabine-capecitabine [8] and the modified fluorouracil, leucovorin, irinotecan, and oxaliplatin (mFOLFIRINOX) regimen [9]. The choice of regimen depends on several factors including patient fitness, recovery from surgery, tolerability of side effects and patient choice. Randomised controlled trials may not always reflect real world outcomes, and frequently include younger patients with more favourable prognostic factors (e.g., low CA19-9) and better fitness, as well as representing a cohort of patients, who by virtue of participating in research, may be inherently more supported and motivated [10]. Moreover, it has been shown that in the United Kingdom there is significant geographical variation in utilisation of adjuvant chemotherapy [11]. In surgical specialties, centralisation of services to high-volume centres aims to reduce variation and improve patient outcomes. However, this remains less common in oncology services, which may explain some of the observed variation [12]. This study aims to assess the real-world outcomes, over a 11-year period, for patients receiving adjuvant chemotherapy for PDAC in the Cheshire and Merseyside Cancer Network, a large centralised surgical and oncology service for Merseyside, Cheshire, North Wales and the Isle of Man (UK). We sought to determine the proportion of patients receiving adjuvant chemotherapy, their tolerance of this post resection and any impact of a failure to complete this treatment in full.

## Methods

### Patients and study design

**Fig. 1:**
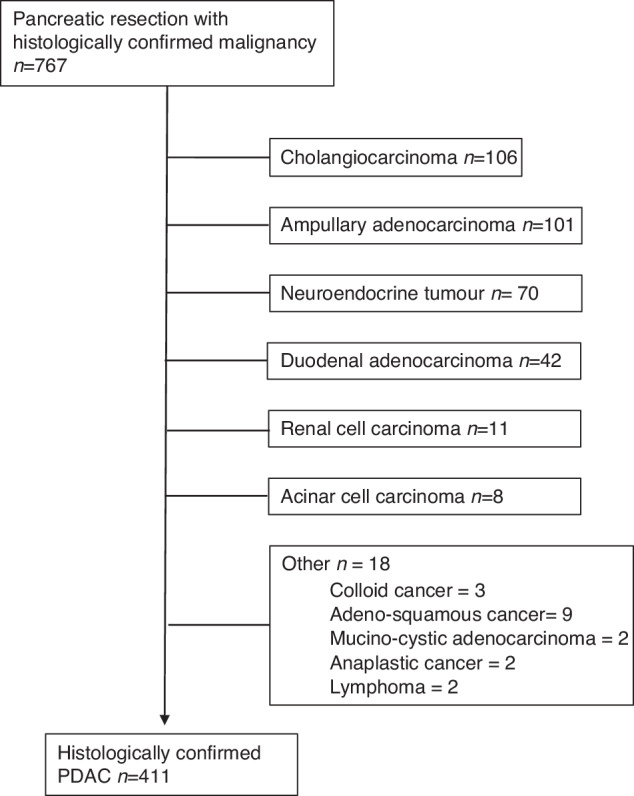
Flowchart of patient selection.

A retrospective review was performed to identify all patients who had undergone a pancreatic resection for a histologically confirmed PDAC at the Royal Liverpool University Hospital (RLUH) between January 2009 and January 2020. Date of referral, histological diagnosis, performance status, site of referral, site of initial review, treatment records and date of death were prospectively collated on a database by Clinical Effectiveness Team (CET) at the Clatterbridge Cancer Centre. Ethical approval to review the database and case records was obtained from the designated committee at the Centre. Patients who had received any form of neoadjuvant treatment were excluded at this stage. Of 767 patients with a histologically confirmed malignancy, only patients with a confirmed PDAC were included for further analysis (Fig. 1). Following patient identification, standard demographic data were obtained from electronic document management systems including age, sex, date of surgery, type of surgery, histopathology, post-operative complications, inpatient/30-day mortality and length of stay (LOS). Post-operative complications were graded using the Clavien-Dindo system [13] with grade 1–2 complications classified as ‘minor’ and grade 3–4 complications classified as ‘major’. Histopathology was reported in accordance with the AJCC TMN 7th edition until 2017 and TMN 8th edition thereafter.

### Selection of patients for adjuvant therapy

Following discharge all patients with a histologically confirmed PDAC were referred to medical oncology for consideration of adjuvant chemotherapy. Since 2013, non-surgical care for pancreatic cancer patients across our cancer network has been centralised to the Clatterbridge Cancer Centre (CCC). This unit is one of the UK’s largest dedicated cancer centres. It provides a comprehensive range of non-surgical cancer therapies, including advanced radiotherapy, chemotherapy, immunotherapy, and molecular targeted therapies. Decisions regarding patient suitability to receive adjuvant chemotherapy were the responsibility of the treating oncology consultant and made following face to face review of patients in clinic and assessment of the patient’s performance status. Where eligible, patients were offered enrolment in relevant adjuvant chemotherapy trials. In addition to the surgical and demographic data, additional data was retrospectively retrieved from oncology records including date of referral to oncology, time since surgery when seen in clinic, chemotherapy regimen, number of cycles, dose reduction and toxicity events. Toxicity was assessed using the National Cancer Institute Common Terminology Criteria for Adverse Events (CTCAE) v5.0.

### Statistical analysis

Analysis was carried out on overall survival (OS) measured from the date of resection to the date of death from any cause. Data collection was commenced in October 2022. This provided a minimum of 2.5 years follow up for all patients from start of adjuvant treatment. Patients alive at start of data collection were censored at this point. Survival estimates were calculated using the Kaplan–Meier method [14]. Log-rank tests were applied to test for evidence of a difference in survival curves between groups. Survival is reported as median (months) with accompanying 95% CI. Survival estimates at 12, 24 and 60 months were also calculated. Multivariable Cox regression modelling was used to assess for independent variables associated with survival. Additional analysis using multivariable logistical regression was performed to identify independent variables associated with a failure to receive adjuvant chemotherapy. Multivariable analyses in each case are performed using a backwards stepwise procedure based on Akaike’s Information Criterion. Following analysis of the full data set, a subgroup analysis was carried out to assess the impact of cycle number and dose reduction on OS using the landmark method [15] by removing from the data any patient who died within 9 months after surgery. This analysis was performed to remove any potential bias as a result of treatment-related deaths and to account for early disease recurrence as a reason for early termination of adjuvant chemotherapy. A *p*-value of <0.05 was considered statistically significant. Statistical analysis was undertaken using R (version 4.0) and Graphpad Prism (version 9.4.1).

## Results

Between January 2009 and January 2020, a total of 411 patients underwent a pancreatic resection for a histologically confirmed PDAC (Supplementary Table 1). Of these patients 46% were female and 54% male. The median age at the time of surgery was 68.2 years (42–85). Most tumours were localised within the pancreatic head with 80% of patients undergoing a pancreatoduodenectomy the majority of which were performed as a pylorus preserving procedure with the remainder undergoing a ‘classical Whipple’. The median length of stay (LOS) post-operatively was 13 days (4–94). As expected, morbidity rates were high in the immediate post-operative period with 45% of patients (185/411) experiencing at least 1 complication of which 24% (45/185) were considered a major (CD grade 3 or grade 4). Despite these complications, 30-day/inpatient mortality was rare, occurring in 10 patients (2.5%). The median overall survival for the entire cohort was 21 months (95% CI 19.4–24.5) with nominal 1-year, 3-year and 5-year survival rates of 73%, 29% and 14% respectively.

### Adjuvant chemotherapy and outcome

**Fig. 2:**
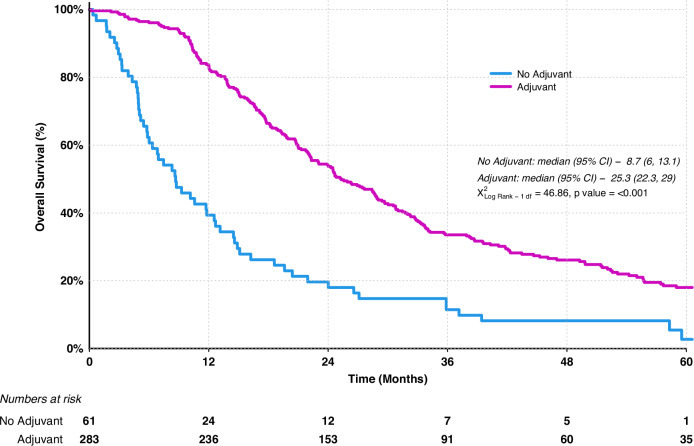
Kaplan–Meier survival analysis of patients who received adjuvant chemotherapy vs no adjuvant chemotherapy following pancreatic resection for histologically confirmed PDAC.

Of the 411 patients included in this study complete oncology records were available in 344. Of these patients 82% (283/344) were confirmed to have commenced adjuvant chemotherapy (Supplementary Table 2) with 67.4% (191/283) completing the intended number of cycles of chemotherapy. The median time between surgery and 1st cycle of chemotherapy for the entire cohort was 9.7 weeks. The most common chemotherapy regimens administered over the course of the study were dual agent gemcitabine-capecitabine (42%) followed by single agent gemcitabine (36%) reflecting the evolution of adjuvant chemotherapy from monotherapy to GemCap doublet and latterly mFOLFIRINOX over the study period, although the centre also participated in the ESPAC3 and ESPAC4 trials during the study period (Supplementary Fig. 1a) [5, 8]. Kaplan–Meier survival analysis confirmed that individuals who commenced adjuvant chemotherapy had a significantly improved OS of 25.25 months (95% CI: 22.31–29.04) versus 8.67 months (95% CI 6.01–13.14) in those that did not (Fig. 2). Multivariate analysis showed receiving adjuvant chemotherapy to be an independent predictor of improved OS (HR 0.31 95% CI 0.21–0.48 *p* = 0.001) (Table 1). Whilst poor tumour grade (HR 1.36 95% CI 1.035–1.778 *p* = 0.0028), ≥T3 stage (HR 2.03 95% CI 1.336–3.096 *p* = 0.001), nodal positivity (HR 2.28 95% CI 1.576, 3.285 *p* = 0.001) and positive R margin (HR 1.82 95% CI 1.27–2.60 *p* = 000.1) were all independent predictors of poor survival. An elevated CA19-9 (>180) before starting adjuvant chemotherapy was associated with worse survival outcomes (HR 1.39 95% CI 1.261, 1.523 *p* = 0.001) (Supplementary Fig. 2). Fourteen percent (14%, 48/344) of patients received their chemotherapy as part of a clinical trial (Supplementary Fig. 3), with the vast majority of these recruited to ESPAC4. We were unable to show any difference in survival depending upon the type of chemotherapy regimen used. Likewise, there was no additional survival benefit for patients who received chemotherapy within 8 weeks of surgery compared to those who started later than 8 weeks. Of the 61 patients that did not receive chemotherapy, 31% (19/61) developed early recurrence, which was presumably progression of micro-metastatic disease present at the time of surgery. The remaining patients failed to receive adjuvant chemotherapy through a combination of patient choice and poor fitness.

**Table 1:** Univariable and multivariable analysis of factors associated with overall survival.

Variable	Median OS months (95% CI)	Univariable HR (95% CI)	*p*	Multivariable HR (95% CI)	*p*
Sex					
Male	22.31 (19.22–27.63)	1			
Female	20.17 (17.77–24.64)	1.07 (0.86–1.32)	0.545		
PS					
0	32.85 (17.77-53.09)	1		1	
1	24.49 (21.88–28.45)	1.18 (0.77–1.81)		1.05 (0.647–1.636)	0.83
2	15.01 (11.97–25.56)	1.92 (1.2–3.09)	0.001	1.7 (1.029–2.804)	0.038
Tumour grade					
Mod	28.98 (24.47–33.02)	1		1	
Poor	16.1 (13.76–19.81)	1.72 (1.39–2.14)	0	1.36 (1.035–1.786)	0.028
T_stage					
½	38.67 (32.23-81.9)	1		1	
¾	19.09 (17.08-22.14)	2.04 (1.5–2.77)	0	2.03 (1.336–3.096)	0.001
N_stage					
Negative	42.25 (35.64-55.65)	1		1	
Positive	18.69 (16.379–21.75)	1.97 (1.49–2.6)	0	2.28 (1.576–3.285)	<0.001
R_margin					
Negative	37.91 (30.03–52.56)	1		1	
Positive	18.2 (16.43–21.94)	1.97 (1.52–2.55)	0	1.82 (1.279–2.603)	0.001
Perineural invasion					
No	38.67 (30.78-NA)	1			
Yes	19.69 (17.64–22.27)	2.26 (1.91–2.5)	0.001		
Lymphovascular invasion					
No	24.16 (20.83–34.23)	1			
Yes	19.81 (17.71–24.01)	1.39 (1.08–1.8)	0.011		
Adjuvant Chemo					
No	8.67 (6.01–13.14)	1		1	
Yes	25.25 (22.31–29.04)	0.4 (0.3–0.53)		0.31 (0.201–0.488)	<0.001
Chemo cycle no. + DR					
≥6 cycles (full dose)	28.5 (24.47–33.54)	1		1	
≥6 cycles (reduced dose)	27.5 (22.04–44.65)	0.88 (0.6–1.28)		1.14 (0.766–1.706)	0.513
<6 cycles (full dose)	12.55 (11.99–31.96)	1.91 (1.18–3.1)		1.66 (1.006–2.744)	0.048
<6 cycles (reduced dose)	11.97 (9.03–NA)	2.44 (1.3–4.57)	0	4.01 (2.006–7.79)	<0.001
Chemotherapy start					
≤8 weeks	25.92 (20.73–33.54)	1			
>8 weeks	25.3 (22.27–29.7)	1.07 (0.78–1.45)	0.674		
Pre chemo CA19-9					
Low	29.86 (26.38–33.34)	1		1	
High	10.96 (9.99–13.76)	3.8 (2.75–5.27)	0	1.39 (1.261–1.523)	<0.001
Chemo regimen					
None	9.71 (6.93–14.59)	1			
Gem	22.17 (18.79–25.3)	0.43 (0.31–0.62)			
GemCap	28.78 (24.28–33.74)	0.3 (0.21–0.43)			
mFOLFIRINOX	28.98 (15.18- NA)	0.28 (0.15-0.54)			
Other	25.92 (20.8–NA)	0.33 (0.17–0.65)	0		

### Peri-operative factors and adjuvant chemotherapy

To identify whether there were any peri-operative factors associated with a failure to receive adjuvant chemotherapy we performed an additional multivariate analysis comparing those individuals who received adjuvant chemotherapy to those that didn’t. There was no difference in age (OR 1.00 95% CI: 0.97–1.06) or gender (OR 0.97 95% CI: 0.34–1.6) between groups. Equally the type of operation performed had no impact, neither did length of post-operative stay (OR 1.03 95% CI: 0.99–1.06 *p* = 0.08). Importantly, the development of a major post-operative complication (CD grade 3/4) had no association with a failure to receive adjuvant chemotherapy (OR 0.46 95% CI: 0.14–1.62 *p* = 0.26). However, patients with a poor performance status (PS 2 or worse) were significant less likely to commence adjuvant treatment (OR 4.16 95% CI: 2.05–8.7 *p* = 0.001) (Table 2).

**Table 2:** Multivariable analysis of operative factors predictive of a failure to receive adjuvant chemotherapy.

Variable	Odds ratio (OR)	95% CI	*p* value
Age	1.007	0.9752–1.044	0.6845
Sex			
Female	1		
Male	0.7368	0.3395–1.584	0.4339
Post-op LOS	1.033	0.9962–1.071	0.0819
Post op complication			
No	1		
Yes	1.235	0.5352–2.827	0.6175
Major complication			
No	1		
Yes	0.461	0.1024–1.624	0.2625
Surgery Performed			
PPPD	1		
Left	2.257	0.8109–5.931	0.1058
Total	0.6874	0.1223–2.763	0.6294
Classical Whipple	0.5507	0.02801–3.411	0.5926
PV resection			
No			
Yes	0.5107	0.1259–1.631	0.2958
PS			
0/1	1		
2	4.169	2.057–8.753	0.0004

### Adjuvant therapy pre- and post-centralisation of medical oncology services

In 2013 medical oncology services for HPB cancers were centralised to the Clatterbridge Cancer Centre (CCC). In the period after centralisation (Supplementary Table 1). there was a notable increase in the proportion of patients that commenced adjuvant treatment (86% vs 69% *p* < 0.05). A Pearson’s Chi-squared test with Yates’ continuity correction was performed to compare chemotherapy completion rates between patients pre and post centralisation. The Chi-squared test revealed no statistically significant difference in chemotherapy completion rates between the two groups (χ²(1) = 0.1761, *p* = 0.67). Although there was an observable trend toward improved OS following centralisation (HR 0.88 (0.71, 1.09), the difference did not reach statistical significance (*p* = 0.223, Supplementary Fig. 4.1). In those patients that received adjuvant chemotherapy, there was no statistically significant difference in OS pre- and post-centralisation (*p* = 0.645, Supplementary Fig. 4.2).

### Impact of cycle number and dose reduction

**Fig. 3:**
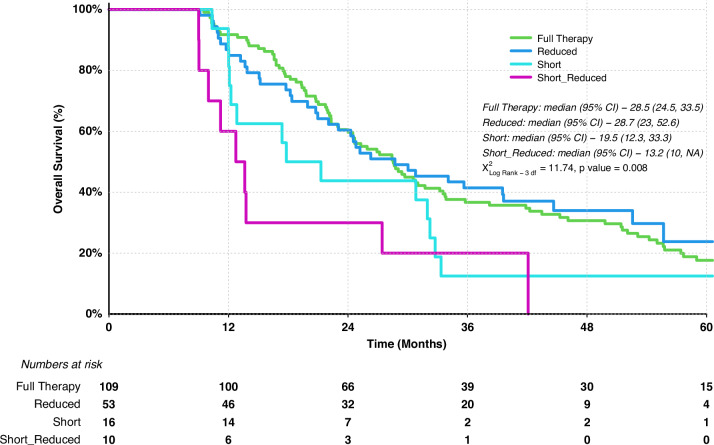
Kaplan–Meier survival analysis using landmark method to assess impact of cycle number and dose reduction on overall survival.

Of the 283 patients that received adjuvant chemotherapy we had complete records regarding cycle number and dose reduction in 207. This enabled us to perform a subgroup analysis of this cohort assessing the impact of cycle number and dose reduction on survival. Out of 207 patients, 164 (79%) received the intended cycles of chemotherapy. Of these patients 54 (33%) required a dose reduction during their treatment course, with the most common being a reduction of 20% (39 patients) followed by 40% (10 patients). Utilising a landmark survival analysis, we showed there to be no survival disadvantage in individuals that completed the full course at a reduced dosage with median survival comparable to those who completed all cycles at the full treatment dose (OS 27.5 months vs. 28.5 months; HR 1.14, 95% CI 0.76–1.70 *p* = 0.513). There was a significant reduction in OS for individuals who received less than the intended number of cycles of chemotherapy (median = 4; IQR 2–5) irrespective of whether this was at a full or reduced dosage (Table 1 and Fig. 3). Of the individuals that failed to complete the intended cycles of adjuvant chemotherapy, 14% ceased treatment due to the development of recurrent disease with the remainder failing to complete therapy through a combination of patient choice (10%), toxicity (28%) and other intercurrent illnesses preventing treatment (15%). In the remaining 32% of patients an explicit reason for stopping chemotherapy was unclear but presumed to be due to a combination of poor tolerance and patient choice. When we adjusted our analysis to exclude those individuals who failed to complete the full number of cycles of chemotherapy due to recurrence, the survival disadvantage from receiving less than full course of chemotherapy persisted (OS 28.8 months vs 13.5 months HR 2.1 95% CI 1.48–3.01 *p* = 0.001).

### Chemotoxicity

68.5% of patients experienced side-effects related to their chemotherapy. In most cases these were mild CTCAE grade 1 (42%) and moderate CTCAE grade 2 (21%) complications. A smaller proportion of patients experienced more significant side-effects with severe grade 3 complications occurring in 4% of patients. Two patients suffered a life-threatening complication. No patient died as a direct result of their adjuvant treatment.

## Discussion

This study shows that real-world survival of patients with PDAC receiving adjuvant chemotherapy is comparable to that published in clinical trials. OS in our patients was 25.25 months (95% CI: 22.31–29.04) which is comparable to that reported in the ESPAC-4 trial with median OS for patients in the gemcitabine-capecitabine group of 28.0 months (95% CI 23.5–31.5) compared with 25.5 months (95% 22.7–27.9) in the gemcitabine only group [8]. This correlation is not surprising considering many of the patients in this study were treated with gemcitabine-based regimens reflecting best practice evidence at the time [5, 8]. More recently the adjuvant treatment landscape has shifted to include combination chemotherapy with mFOLFIRINOX. The PRODIGE-24 trial reported a median OS of 54.4 months in the mFOLFIRINOX group with a nominal 3-year survival of 63.4%, albeit at the cost of significantly increased risk of grade 3 and 4 adverse events [9]. Only a small proportion of our patient cohort in 2019 received mFOLFIRINOX (*n* = 20) following publication of the PRODIGE trial in December 2018. As expected, most were physically fit non-smokers with a good performance status. Fourteen patients (70%) managed to complete the full course of treatment. However, 75% reported toxicities during their treatment with the majority (60%) requiring a dose reduction. Due to this limited patient number, we were unable to make an accurate assessment of survival and further study in a larger patient cohort with sufficient follow up is required.

Despite the well-established role of adjuvant chemotherapy in improving survival outcomes, significant variation in its delivery rates persists across the UK. In this study 82% of our treatment population commenced adjuvant treatment. Adjuvant chemotherapy delivery rates in England are higher than in Europe and the USA [11, 16, 17] which may be partly attributable to the involvement of UK institutions in adjuvant chemotherapy clinical trials. Whilst most UK centres report adjuvant chemotherapy delivery rates between 40.7% and 64.5%, some centres achieve significantly higher rates. A survival analysis of all patients pre- and post-centralisation showed an observable trend toward improved overall survival following centralisation (HR 0.88 (0.71, 1.09); however, the difference did not reach statistical significance (Supplementary Fig. 4.1). Translating increased delivery of adjuvant therapy into improved overall survival also relies heavily on early detection, optimal surgery, improved access to supportive care, and enhanced patient fitness. While the delivery of adjuvant therapy is integral to outcomes, significantly improving survival in this setting requires addressing multiple contributing factors in parallel. Despite a more heterogeneous patient population receiving adjuvant treatment post-centralisation, survival was not found to decrease (Supplementary Fig. 4.2). This suggests that centralisation of care, with an increased focus on holistic patient management and support throughout treatment, is effective. Apart from poor performance status, we were unable to identify any factors associated with a failure to commence adjuvant chemotherapy. Patients who experienced a significant post-operative complication (CD grade 3 and 4) did not appear disadvantaged. This finding is consistent with that reported in the ESPAC1, ESPAC3 and ESPAC4 trials as well as the PANASTA trial which showed patients with post-operative complications including clinically relevant post-operative pancreatic fistula were no less likely to receive adjuvant chemotherapy and had comparable survival outcomes [5, 7, 8, 18].

The centralisation of pancreatic surgery into high volume centres is known to improve surgical outcomes and reduce variation between units [19]. Where at all possible, consideration should be given to a similar arrangement for the delivery of HPB oncology services in a bid to increase delivery rates. In our region the centralisation of HPB medical oncology services occurred in 2013 and resulted in an increase in the proportion of patients receiving adjuvant treatment. This reflects our previous experience in those requiring treatment for advanced disease [20]. The increase in adjuvant chemotherapy delivery observed following centralisation of oncology services, as shown in this study, is likely to be multifactorial. The consolidation of services into specialist centres creates hubs of greater clinical expertise and experience, with access to multidisciplinary support services, including dietitians, physiotherapists, clinical psychologists, and oncology clinical nurse specialists, which are crucial for optimising post-operative recovery and treatment readiness. These environments facilitate coordinated post-operative review processes, allowing early identification of suitable patients and prompt initiation of supportive treatments. This ensures that patients who are initially of borderline fitness or experience post-operative complications are actively managed and optimised for treatment, rather than excluded after initial review. Moreover, enhanced access to key supportive care services, such as rapid dietetic assessment, and access to immediate enhanced supportive care, has the potential to improve performance status, particularly relevant in pancreatic cancer where post-operative morbidity and malnutrition frequently delay or preclude chemotherapy. Specialist oncology nursing input also plays a significant role in quickly addressing physical, psychological, and social barriers to treatment, improving patients’ ability to commence systemic therapy within the limited post-operative window available. Importantly, centralisation enables these issues to be assessed and managed comprehensively at, or even before, the first oncology review. The cumulative effect appears to be that more patients are assessed in a timely, structured manner and supported to initiate adjuvant chemotherapy. While centralisation of treatment may require some patients to travel further for care, the demonstrable benefits of receiving care in high-volume, specialist centres—including higher rates of adjuvant therapy delivery—appear to outweigh these logistical challenges for many patients. However, we acknowledge that this may not be the case for everyone, and this should be discussed with patients, addressing barriers where possible. Comparable improvements in outcomes associated with service centralisation have also been reported in other cancer types, supporting the broader value of this model of care [21–24].

The completion of treatment is particularly important as our data shows a significant determinant of outcome is successfully completing the full course of chemotherapy, a finding previously reported from the ESPAC3 and PRODIGE24 trial data [9, 25]. We show this survival benefit to persist irrespective of whether a dose reduction is required to complete the full treatment course. This suggests that it is cycle number rather than dose intensity that is most important in determining outcome and greater consideration should be given to altering a patient’s dosing regimen to ensure they can complete the maximum number of cycles possible. Our study also showed that timing of chemotherapy did not influence OS with no difference in outcome if chemotherapy is commenced beyond 8 weeks of surgery. This suggests patients should be given adequate time to recover their fitness post-operatively and reassessed prior to making a definitive treatment decision. As shown in previous studies, we also noted that an elevated CA19-9 in the post-operative period prior to adjuvant treatment was a strong predictor of OS and remains an important investigation post-resection, as it has the potential to guide decision making in the adjuvant setting [26, 27].

Our study has certain limitations. It is retrospective in nature, with inherent biases that this design can introduce. Observational data collected over a 11-year period presents challenges, and we acknowledge that concurrent patient cohorts would have been the ideal approach. However, selection bias was minimized by including all consecutive resections for PDAC. Complete oncology data, particularly regarding cycle numbers and dosing, were limited, especially for patients treated outside the Mersey region (Wales/Isle of Man), where we relied heavily on clinician documentation. Despite these limitations, we believe our study provides an accurate reflection of real-world survival data and adjuvant chemotherapy delivery rates in a PDAC cohort.

## Conclusion

This study shows that real-world survival in patients receiving adjuvant chemotherapy is comparable to that reported in clinical trials. Over 80% of our patients commenced adjuvant treatment showing it is possible to achieve high delivery rates following complex surgical intervention. However, it is imperative that we optimise and support patients fully to ensure patients are given the opportunity to complete full course of chemotherapy, as this is suggested to be associated with a significant survival benefit, even if a dose reduction is required to achieve this. To help deliver on both these objectives and thereby improve patient outcomes a greater consideration should be given to the centralisation of HPB oncology services, ensuring cohesive and holistic patient care.

## Supplementary information

Supplementary material

## Data Availability

Data underlying this article were collected as part of a local clinical audit of outcomes for patients treated for pancreatic cancer at The Clatterbridge Cancer Centre, Liverpool. Due to patient confidentiality and NHS/local information governance requirements, the dataset is not publicly available. Anonymised data may be made available from the corresponding author upon reasonable request, subject to institutional approval.
