# Supplementary material for: Real-world outcomes following adjuvant chemotherapy for resected pancreatic cancer in a centralised oncology service
Source: Br J Cancer. 2026 Feb 25;134(8):1183–9. doi: 10.1038/s41416-026-03341-0 (PMC13036073; doi:10.1038/s41416-026-03341-0)
Supplement: Supplementary file 1 — Supplementary material [file 41416_2026_3341_MOESM1_ESM.docx]

|  | **Pre-Centralisation**  **(2009-2012)** | **Post Centralisation**  **(2013-2019)** | **Total** |
| --- | --- | --- | --- |
| **Total number of patients referred for consideration of chemotherapy** | 120 | 291 | 411 |
| **Chemotherapy received (%)**  No  Yes  - Completed treatment  Unknown | 25 (31)  56 (69)  36  39 | 36 (14)  227 (86)  155  28 | 61 (18)  283 (82)  191  67 |
| **Median Age (range)** | 66 (39-83) | 69 (41-85) | 68.2 (42-85) |
| **Sex – no. (%)**  Male  Female | 65 (54)  55 (46) | 155 (53)  136 (47) | 220 (54)  191 (46) |
| **PS**  0  1  2  3  unknown | 4 (3)  39 (33)  11 (9)  2 (2)  64 (53) | 27 (9)  147 (51)  47 (16)  1 (0.3)  69 (23.7) | 31 (8)  186 (45)  58 (14)  3 (1)  133 (32) |
| **CCI (%)**  0  1  2  3  4  5  N/A | 0 (0)  4 (3)  12 (10)  8 (7)  6 (5)  1 (1)  89 (74) | 9 (3)  36 (12)  51 (18)  72 (25)  33 (11)  14 (5)  76 (26) | 9 (2)  40 (10)  63 (15)  80 (19)  39 (10)  15 (4)  165 (40) |
| **Operation (%)**  PPPD  Classic Whipple (KWPD)  Total  Left | 97 (80)  7 (6)  10 (9)  6 (5) | 208 (71.5)  18 (6)  19 (6.5)  46 (16) | 305 (74)  25 (6)  29 (7)  52 (13) |
| **Post op Complication (%)**  No  Yes  Major (CD grade 3&4)  Death (CD grade 5)  Unknown | 59 (49)  50 (42)  11  4  11 (9) | 156 (53.5)  135 (46.5)  34  6  0 (0) | 215 (52.5)  185 (45)  45  10  11 (2.5) |
| **T stage – no. (%)**  1  2  3  4 | 3 (2.5)  4 (3.5)  112 (93)  1 (<1) | 16 (5.5)  55 (19)  215 (74)  5 (1.5) | 19 (4.5)  59 (14.5)  327 (79.5)  6 (1.5) |
| **Nodal status – no. (%)**  0  1  2 | 21 (18)  99 (82)  0 (0) | 69 (24)  193 (66)  29 (10) | 90 (22)  292 (71)  29 (7) |
| **Resection margin (%)**  Negative  Positive | 29 (24)  91 (76) | 76 (26)  215(74) | 105 (25.5)  306 (74.5) |
| **Tumour grade (%)**  Well differentiated  Moderately differentiated  Poorly differentiated | 12 (10)  64 (53)  44 (37) | 5 (1.5)  144 (49.5)  142 (49) | 17 (4)  208 (51)  186 (45) |
| **Lymphovascular invasion (%)**  No  Yes  Unknown | 35 (29)  72 (60)  13 (11) | 67 (23)  224(77)  0 (0) | 102 (25)  296 (72)  13 (3) |
| **Perineural invasion (%)**  No  Yes  Unknown | 14 (11.5)  93 (77.5)  13 (11) | 20 (7)  271(93)  0 (0) | 34 (8)  364 (89)  13 (3) |
| **Median CA19.9 <3 months post resection** | 34.50 | 35.50 |  |

**Supplementary table 1:** Patient demographics, stratified pre and post centralisation

|  | **Adjuvant therapy**  **(n= 283)** | **No adjuvant therapy**  **(n=61)** |
| --- | --- | --- |
| **Sex (%)**  Male  Female | 150 (53)  133 (47) | 30 (49)  31 (51) |
| **Age (Y)** | 67 (39-84) | 72 (41-85) |
| **PS**  0  1  2  3  Unknown | 31  168  40  1  43 | 0  18  18  2  23 |
| **Comorbidities**  None  Mild  Moderate  Severe  unknown | 8  96  102  12  65 | 1  6  17  3  34 |
| **Operation performed (%)**  PPPD-1  KW-PD-2  Total-3  Left-4 | 219 (77)  16 (6)  19 (7)  29 (10) | 41 (67)  1 (2)  5 (8)  14 (23) |
| **LOS (days)** | 12 (10-16) | 17 (14-25) |
| **Post op Complication (%)**  No  Yes  Major (CD grade 3&4 | 162 (57)  121 (43)  35 | 24 (39)  37 (61)  11 |
| **Tumour grade (%)**  Well diff  Mod diff  Poorly diff | 14 (5)  148 (52)  121 (43) | 2 (3)  21 (35)  38 (62) |
| **T stage (%)**  I  II  III  IV | 14 (5)  40 (14)  226 (80)  3 (1) | 3 (5)  3 (5)  52 (85)  3 (5) |
| **Lymph nodes (%)**  negative  positive | 61 (22)  222 (78) | 10 (16)  51 (84) |
| **R status (%)**  negative  positive | 79 (28)  204 (72) | 12 (20)  49 (80) |
| **Perineural invasion**  Yes  No  Unknown | 260  21  2 | 6  54  1 |
| **Lymphovascular invasion**  Yes  No  Unknown | 213  65  5 | 46  14  1 |
| **Postop Ca 19.9**  Median | 31 (1-5930) | 97(10-234996) |

**Supplementary Table 2**- Patient and tumour characteristics of patients stratified by whether patients received adjuvant chemotherapy or not.


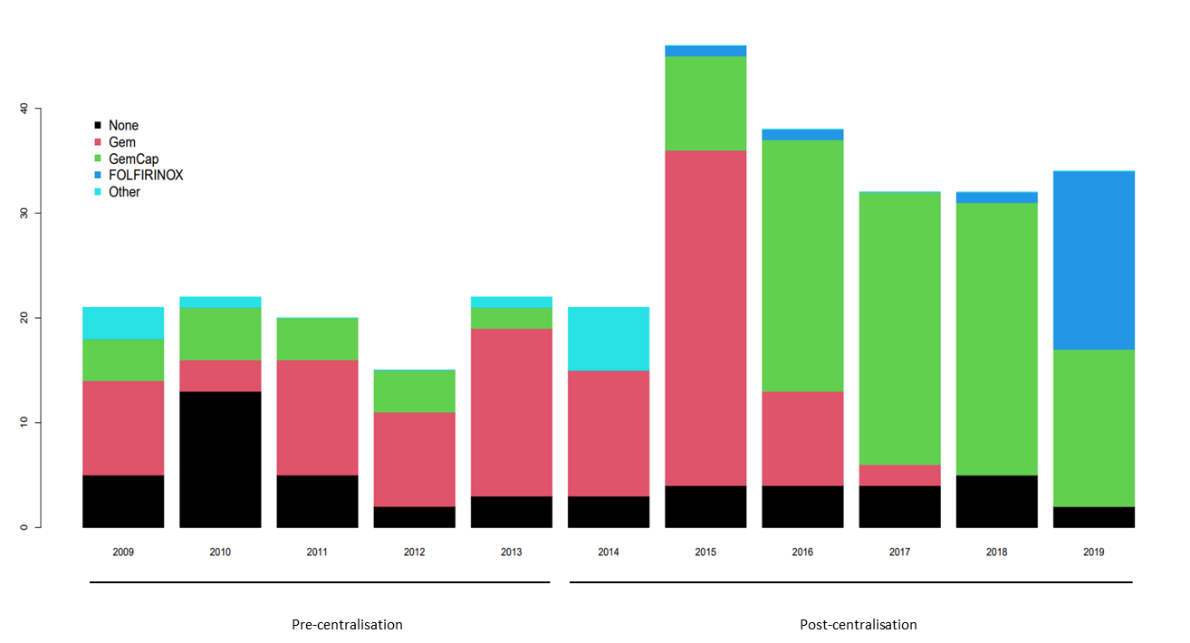


**Supplementary Figure 1a**: Frequency of patients receiving adjuvant treatment and type of chemotherapy regimen delivered per year of study

**Supplementary Figure 1b**: Proportion of patients receiving full course (full dose and all planned cycles), reduced course (reduced dose, full course), short (full dose, reduced course) short and reduced course (reduced dose, reduced course length).

**Supplementary Figure 2**: Kaplan-Meier survival analysis of patient with elevated post-operative CA19-9 prior to delivery of adjuvant chemotherapy.

Supp figure 3: a) Kaplan-Meier survival analysis of patients receiving adjuvant chemotherapy on or off trial.

**Supplementary Figure 4.1**: a) Kaplan-Meier survival analysis of all patients (adjuvant and no adjuvant treatment) pre- and post-centralisation comparison, HR 0.88 (0.71, 1.09)

**Supplementary Figure 4.2**: Kaplan-Meier survival analysis pre and post centralisation for those patients who received adjuvant received chemotherapy (n=283).

**** **Supplementary Figure 5:** Kaplan-Meier survival analysis comparing individual chemotherapy treatments
